# ZEB1-AS1 initiates a miRNA-mediated ceRNA network to facilitate gastric cancer progression

**DOI:** 10.1186/s12935-019-0742-0

**Published:** 2019-02-06

**Authors:** Ming-Hui Ma, Jia-Xiang An, Cheng Zhang, Jie Liu, Yu Liang, Chun-Dong Zhang, Zhen Zhang, Dong-Qiu Dai

**Affiliations:** 1 Department of Gastroenterological Surgery, The Fourth Affiliated Hospital of China Medical University, Shenyang, 110032 China; 2 Science Experiment Center, China Medical University, Shenyang, 110122 China

**Keywords:** Gastric cancer, Biomarker, ZEB1-AS1, miR-149-3p, GC progression, ceRNA regulatory network

## Abstract

**Background:**

Currently, cancer-related competing endogenous RNA (ceRNA) networks are attracting significant interest. As long noncoding RNA ZEB1-AS1 has been reported to function as an oncogene due to sponging microRNAs (miRNAs) in several cancers, we hypothesized that it could interact with specific miRNAs to form regulatory networks and facilitate the growth of gastric cancer (GC).

**Methods:**

MiRNAs interacting with ZEB1-AS1 were screened for and selected by bioinformatics analysis. Overexpression or repression of ZEB1-AS1 was performed to determine whether it could regulate selected miRNAs. Quantitative real-time polymerase chain reactions (qPCR) validated the expression profiles of ZEB1-AS1 and miR-149-3p in GC cell lines and tissue. Statistical analysis determined the clinical significance of ZEB1-AS1 in relation to miR-149-3p. Cell counting, wound healing and transwell assays were performed to assess cell proliferation, migration and invasion. A luciferase reporter assay was utilized to confirm the putative miR-149-3p-binding sites in ZEB1-AS1.

**Results:**

Briefly, bioinformatics analysis inferred that ZEB1-AS1 interacts with miR-204, miR-610, and miR-149. Gain- or loss-of function assays suggested that ZEB1-AS1 negatively regulates miR-149-3p, miR-204-5p and miR-610 in GC cells. Validated by qPCR, ZEB1-AS1 was up-regulated and miR-149-3p down-regulated in GC cells and tissue. Data analyses indicated that ZEB1-AS1 and miR-149-3p are associated with the independent diagnosis and prognosis of GC. Functional assays support the theory that miR-149-3p hinders GC proliferation, migration and invasion, whereas its overexpression abrogates the corresponding effects induced by ZEB1-AS1. Lastly, dissection of the molecular mechanisms involved indicated that ZEB1-AS1 can regulate GC partly via a ZEB1-AS1/miR-149-3p axis.

**Conclusions:**

ZEB1-AS1 can interact with specific miRNAs, forming a miRNA-mediated ceRNA network and promoting GC progress, partly through a ZEB1-AS1/miR-149-3p axis.

**Electronic supplementary material:**

The online version of this article (10.1186/s12935-019-0742-0) contains supplementary material, which is available to authorized users.

## Background

Gastric cancer (GC) is a hazard to public health, causing the second largest number of cancer-related deaths globally [1]. Eastern Asian populations are particularly susceptible compared to other world populations [2]. Due to early diagnosis and comprehensive treatment strategies, which include chemotherapy, radiotherapy, targeted therapy and surgical intervention, the overall survival rate has improved slightly [3, 4]. However, patients in advanced stages, who often appear with local infiltration, refractory proliferation, and even distant metastasis, might encounter poor outcomes [5]. Therefore, it is of significance to investigate the underlying mechanism(s) of GC initiation and progression, as well as to identify effective biomarkers that are indicative of GC diagnosis and prognosis.

Long noncoding RNAs (lncRNAs) are a group of transcripts that are over 200 nucleotides in length. Formerly considered to be “transcriptional junk”, lncRNAs are currently considered as “novel regulators” in various cancers. They can coordinate gene expression by acting as decoys for transcriptional factors, scaffolds for chromatin modifying complexes, or compete with other genomic elements in binding to miRNAs [6–8], and play crucial roles in the development, progression, invasion and metastasis of multiple cancers [9–11]. Additionally, lncRNAs serve as valuable biomarkers for cancer diagnosis and prognosis [12–14]. LncRNA ZEB1 antisense 1 (ZEB1-AS1) is transcribed from a shared bi-directional promoter of the zinc finger E-box binding homeobox 1 gene (ZEB1). An increasing number of studies have provided evidence that ZEB1-AS1 has oncogenic properties and serves as a promising biomarker in multiple cancers; GC included. For example, ZEB1-AS1 activates prostate cancer by regulating ZEB1 and the expression of the downstream molecule [15]. It has also been associated with predictions of unfavorable prognoses and the promotion of tumor metastasis in hepatocellular cancer [16] and gliomas [17], and is further associated with the progression of esophageal squamous cell carcinoma and patient survival [18]. In GC, ZEB1-AS1 plays a cancer-promoting role and is related to poor prognosis [19], while in osteosarcoma, ZEB1-AS1 epigenetically activates ZEB1 to facilitate tumor progression [20]. Nevertheless, the regulatory mechanism involved in GC and the prospect of the clinical application of ZEB1-AS1 in identifying GC is still limited and warrants further investigation.

Another type of noncoding RNA, microRNAs (miRNAs), are 19–25 nucleotides in length, are well characterized, and primarily participate in gene regulation via mRNA transcript degradation or translation inhibition. They dominate biological behaviors, such as proliferation, migration, invasion, and apoptosis, of tumor cells [21, 22]. A considerable number of miRNAs are critical regulatory elements that facilitate or suppress GC occurrence and progression and might serve as therapeutic targets and novel biomarkers for GC [23–26].

Recently, some studies suggested that lncRNAs engage in crosstalk with mRNAs and act as competing endogenous RNAs (ceRNAs) by sequestering shared miRNAs [8, 27–29]. It has been suggested that lncRNAs, mRNAs and miRNAs communicate with each other and form competitive endogenous networks, which could be of profound significance to tumor mechanism investigations. For instance, lncRNA-KRTAP5-AS1 and lncRNA-TUBB2A regulate CLDN4 and influence tumor formation and metastasis via a ceRNA-mediated regulatory network in GC [30], while HOTTIP promotes small cell lung cancer via a HOTTIP/miR-574-5p/EZH1-associated ceRNA network [31]. Several studies have documented that ZEB1-AS1 can aggravate malignant behaviors of cancer cells through miRNA-mediated mechanisms [32–34]. However, it is still uncertain whether ZEB1-AS1 can interact with miRNAs to form a ceRNA network in GC.

In the present study, significantly down-regulated miRNAs predicted to bind to ZEB1-AS1 were selected by bioinformatics analysis and included miR-204, miR-610, miR-149. Subsequently, it was found that ZEB1-AS1 negatively modulates miR-204-5p, miR-610, and miR-149-3p in vitro. Furthermore, we evaluated the functional effects of miR-149-3p on GC cell proliferation, migration, and invasion and suggest the existence of a ZEB1-AS1/miR-149-3p axis. Collectively, ZEB1-AS1 can interact with specific miRNAs, form a ceRNA regulatory network, and, in part, promote GC progression through a ZEB1-AS1/miR-149-3p axis.

## Materials and methods

### ZEB1-AS1-related miRNAs mining and integrated analysis

The miRNA expression profiles of GC were downloaded from the TCGA database (https://cancergenome.nih.gov/) and processed to screen for differentially expressed miRNAs with the limma package in R. Subsequently, RNA22 version 2.0 software (https://www.rna-seqblog.com/rna22-version-2-0-mirna-mre-predictions/) was employed to detect the miRNAs binding to ZEB1-AS1. Thus, miRNAs that were both down-regulated and could bind to ZEB1-AS1 were identified and included miR-204, miR-610, and miR-149. Three online websites, TargetScan (http://www.targetscan.org/), miRDB (http://www.mirdb.org/miRDB/) and DIANA (http://www.microrna.gr/microTCDS) were then utilized to predict the potential target genes of the three miRNAs. The most commonly targeted genes from the three websites were obtained by Venn diagrams. Functional gene enrichment analysis was performed through the Visualization and Integrated Discovery (DAVID) tool (http://david.abcc.ncifcrf.gov/) to elucidate the biological function of the common target genes [35].

### Tissue samples and cell lines

Eighty-four fresh GC tissue samples from patients who received surgical re-sectioning between January 2010 and October 2012, together with 47 precancerous gastric lesions (dysplasia) and 59 healthy gastric mucosal tissue samples from volunteers who underwent pathological biopsy under gastroscopy, were provided by the China Medical University Cancer Institute (Shenyang, China). Each specimen was frozen at − 80 °C in liquid nitrogen until studied. Every participant provided informed consent, with surgical patients subjected to post-operative follow-up for 65 months. The study was carried out under the approval of the China Medical University ethics committee in accordance with the Helsinki Declaration (1975). Clinicopathological data involved in the study is listed in Table 1. The Chinese Academy of Sciences Cell Bank (Shanghai, China) provided the cell lines used in the study, which included a normal human gastric cell line (GES-1) and five gastric cancer cell lines (SGC-7901, MGC-803, MKN-45, HGC-27, and AGS). All cells were cultured in RPMI 1640 medium (HyClone; GE Healthcare Life Sciences, Logan, UT, USA) supplemented with 10% fetal bovine serum (HyClone; HyClone Laboratories Inc. Victoria, Australia), 100 U/mL penicillin and 100 µg/mL streptomycin. The cell growth conditions were maintained at a constant temperature of 37 °C with 5% CO_2_ and invariable humidity.

**Table 1:** Correlation between clinicopathological characteristics and ZEB1-AS1 or miR-149-3p

Parameter	Case number	ZEB1-AS1 expression		P-value	miR-149-3p expression		P-value
		High (n = 42)	Low (n = 42)		High (n = 42)	Low (n = 42)	
Age (years)				0.507			0.268
< 60	35	19	16		20	15	
≥ 60	49	23	26		22	27	
Gender				0.124			0.826
Male	47	27	20		24	23	
Female	37	15	22		18	19	
Tumor size (cm)				0.02*			0.188
≤ 5	38	12	26		22	16	
> 5	46	30	16		20	26	
Differentiation				0.127			0.275
Well/moderate	43	18	25		24	19	
Poor	41	24	17		18	23	
Invasion depth				0.387			0.027*
T1–T2	36	16	20		23	13	
T3–T4	48	26	22		19	29	
Lymph node metastasis				0.01*			0.126
No	39	12	27		23	16	
Yes	45	30	15		19	26	
TNM stage				0.048*			0.04*
I–II	37	14	23		25	12	
III–IV	47	28	19		17	30	
CA-199 (tissue) (U/mL)				0.825			0.268
< 37	35	17	18		20	15	
> 37	49	25	24		22	27	

*n* number; *y* year

* P < 0.05

### Cell transfection

For the establishment of stably transfected cells, lentiviruses carrying ZEB1-AS1 and ZEB1-AS1-sh-RNA vectors were constructed (Wanleibio, Shenyang, China) and respectively transfected into SGC-7901 and MGC-803 cells according to the manufacturer’s protocol. Following 48 h of transfection, cells were selected with 10 μg/mL puromycin, measured through a fluorescent inverted microscope, with qPCR performed to determine the transfection efficiency (Additional file 1: Fig. S1). The miR-149-3p mimic and the miR-149-3p negative control (miR-NC) elements (GenePharma Corporation, Shanghai, China) were transiently and separately transfected into the same cell lines using Lipofectamine TM 3000 transfection reagent (Invitrogen, Carlsbad, CA, USA). Cells and transfection complexes were co-incubated for 5 h. Transfection efficiency in these cells was also validated by qPCR. The sequences involved in the study are detailed in Additional file 2: Table S1 and Additional file 3.

### RNA isolation, cDNA synthesis and real-time PCR

Total RNA was isolated from frozen samples and cell lines with RNAiso Plus reagent (TaKaRa, Dalian, China) and complementary DNA (cDNA) generated from reverse transcription of 1 µg of total RNA using PrimeScript RT reagent Kit with gDNA Eraser (TaKaRa). Real-time PCR was performed with SYBR^®^ Premix Ex TaqTM kit (TaKaRa) on an ABI 7500 system (Applied Biosystems, Foster City, CA, USA), according to the following PCR conditions: 30 s at 95 °C for initial denaturation, followed by 45 cycles of 5 s at 95 °C for denaturation, 10 s 60 °C for annealing and 30 s 72 °C for extension. For miRNA detection, reverse transcription was accomplished with miRcute plus miRNA First-Strand cDNA Synthesis Kit (Tiangen, Beijing, China). Real-time PCR was conducted with a miRcute plus miRNA qPCR Detection Kit (Tiangen). The primer sequences are shown in Additional file 2: Table S1. RNA expression was normalized to U6 or GAPDH; relative RNA expression was calculated through the 2^−ΔΔCt^ method.

### Cell proliferation assay

The CCK-8 (Cell Counting Kit-8) assay was performed to detect cell viability following the manufacturer’s instructions (Dojindo Laboratories, Kumamoto, Japan). In brief, 4 × 10^3^ transfected cells were seeded into 96-well plates in quintuplicate and incubated for 24, 48, 72, and 96 h. Then, 10 μL CCK8 working solution was added to the medium for 4 h at the fixed time points. Absorbances were measured on a microplate reader at 450 nm. The experiment was performed in triplicate.

### Wound healing assay

The transfected cells were seeded separately into six-well plates (5 × 10^5^ cells/well) and cultured overnight. Once a monolayer of cells had formed, a 200-μL pipette tip was used to scratch the cell layer. After cells present in suspension and cell debris were washed out with PBS, cells were cultured in serum-free medium to permit wound healing. Phase-contrast images were taken at the same position under an inverted microscope at 0, 24, and 48 h after scratching. Three independent experiments were conducted.

### Transwell migration and invasion assays

For the transwell assay, transwell chambers with 8-μm porous membranes (Corning, NY, USA) were used. A total of 5 × 10^4^ transfected cells were added with 200 μL serum-free medium to the upper chambers, the membranes of which were pre-coated with Matrigel (BD Bioscience, San Jose, CA, USA) for invasion assays but uncoated when performing migration assays. A volume of 750 μL medium containing 10% FBS was added to the lower chamber. Following at least 24 h of incubation at 37 °C with 5% CO_2_, upper-chamber cells were removed using a cotton swab. Cells traversing the membranes were fixed in 4% paraformaldehyde and stained with 0.1% crystal violet for 20 min. Cell counts were completed with at least five random visual fields conducted per membrane under a light microscope. The assays were performed in triplicate.

### Luciferase reporter assay

The miR-149-3p-binding sites in ZEB1-AS1 (ZEB1-AS1 wild type or wtZEB1-AS1) and the corresponding mutant sites (ZEB1-AS1 mutant type or mutZEB1-AS1) were respectively amplified by PCR and cloned into the pmirGLO plasmid (Promega, Madison, WI, USA) which contains a luciferase gene. Consequently, miR-149-3p mimics or miR-NC were co-transfected with luciferase reporter plasmids into 293T cells. Luciferase activity was analyzed 48 h post-transfection using the Dual-Luciferase Reporter Assay System (Promega) and normalized against Renilla luciferase activity.

### Statistical analysis

SPSS 13.0 (SPSS Inc, Chicago, IL, USA) was used for statistical analyses. A X^2^ test was utilized to assess categorical variables with Student’s t-tests and analysis of variance (ANOVA) carried out for appropriate comparisons. The Kaplan–Meier method with a log-rank test was adopted to analyze overall survival (OS). Prognostic factors were evaluated using Cox regression analysis. The diagnostic value of each biomarker was tested via receiver operating characteristic (ROC) curve analysis. Spearman’s correlation analysis was applied to examine the correlation between ZEB1-AS1 and miR-149-3p. P-values less than 0.05 were considered statistically significant.

## Results

### Prediction of the dysregulated miRNAs by bioinformatics analysis

**Fig. 1:**
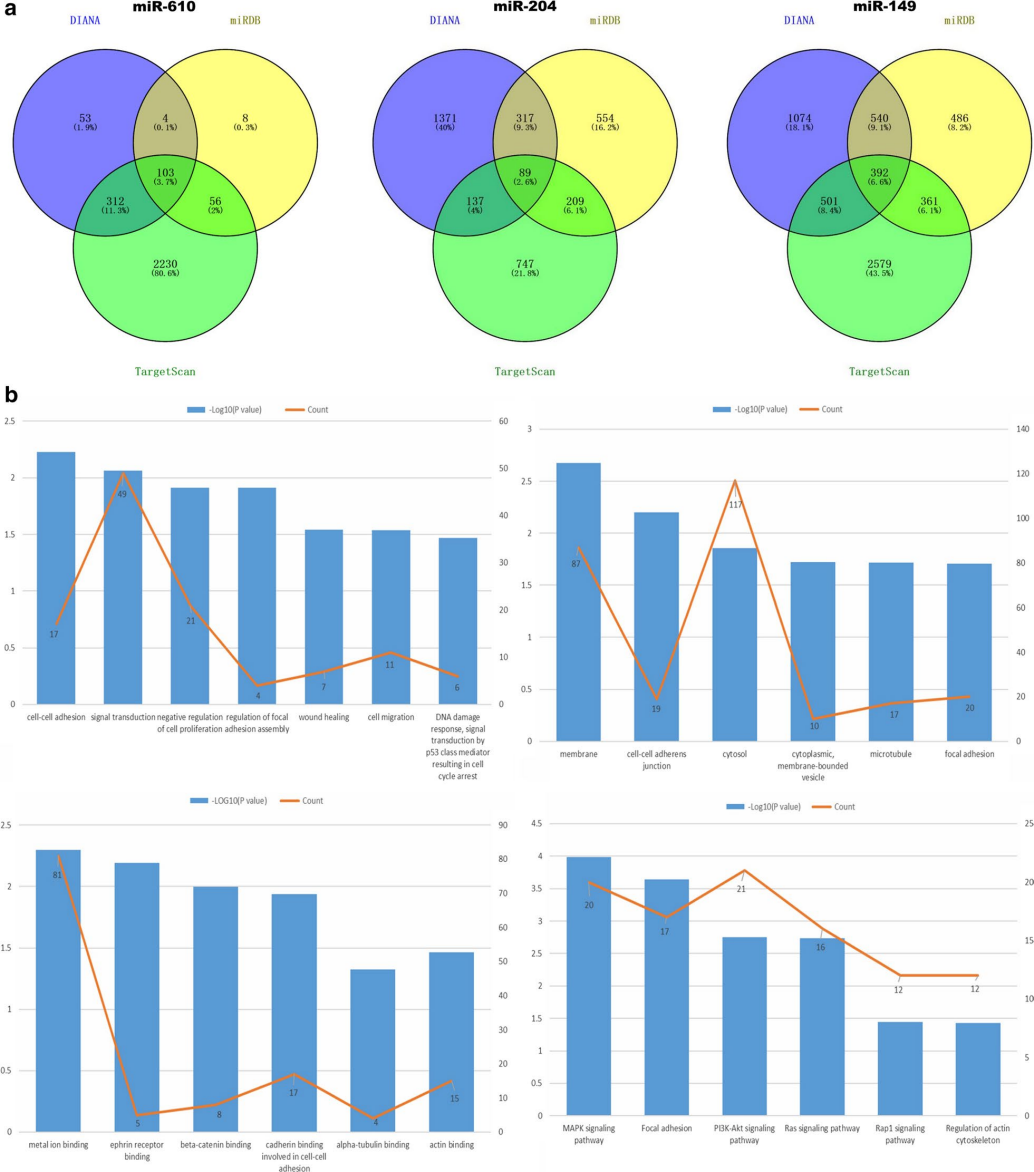
Target gene prediction and function analysis. **a** The common genes targeted by the three miRNAs as predicted by DIANA, miRDB, and TargetScan. **b** Biological process analysis (upper left) indicated that the genes are associated with cell–cell adhesion, signal transduction, negative regulation of cell proliferation, regulation of focal adhesion assembly, wound healing, and cell migration. Cellular component analysis (upper right) showed that the genes are associated with cell–cell adherent junctions, membranes, focal adhesion, cytosol, and microtubules. Molecular function analysis (lower left) indicated that the genes are involved in metal ion binding, cadherin binding cell–cell adhesion, and beta-catenin binding. Biological pathway analysis (lower right) revealed that the genes participate in PI3K-Akt, MAPK, and Ras signaling pathways

Conforming to cut-off criteria of fold changes > 2.0 and P < 0.05, 36 down-regulated miRNAs were obtained by screening the data from 375 GC and 32 adjacent normal tissue samples (Additional file 4). Using RNA22 (version 2.0), 777 miRNAs potentially binding to ZEB1-AS1 were identified (Additional file 5). By intersecting the two miRNA groups, eight down-regulated miRNAs (miR-610, miR-6510, miR-6499, miR-149, miR-4648, miR-6778, miR-204, and miR-770) were selected (Additional file 6). Current published literature has indicated that miR-610, miR-149 and miR-204 can act as tumor suppressors in multiple cancers, including GC [36–38] and colorectal cancer [39]. Hence, these three miRNAs were chosen for subsequent study. Additionally, by applying TargetScan, miRDB and DIANA, a total of 584 consensus genes targeted by the three miRNAs were acquired (Fig. 1a). After removing eight repetitive genes, the remaining 576 target genes (Additional file 7) underwent gene enrichment analysis in order to evaluate the biological functions of the common target genes (Fig. 1b). Biological process analysis revealed that the genes were mainly enriched in cell–cell adhesion, signal transduction, the negative regulation of cell proliferation, focal adhesion assembly regulation, wound healing, and cell migration processes. Cellular component analysis revealed that the genes were mostly enriched in cell–cell adherence junctions, membranes, focal adhesion molecules, cytosol, and microtubules. Molecular functional analysis indicated that the genes were enriched in the binding of metal ions, cadherin binding in cell–cell adhesion, and beta-catenin binding. Biological pathways were mainly enriched for the PI3K-Akt, MAPK, and Ras signaling pathways (Additional file 8).

### Repression of the miRNAs by ZEB1-AS1

**Fig. 2:**
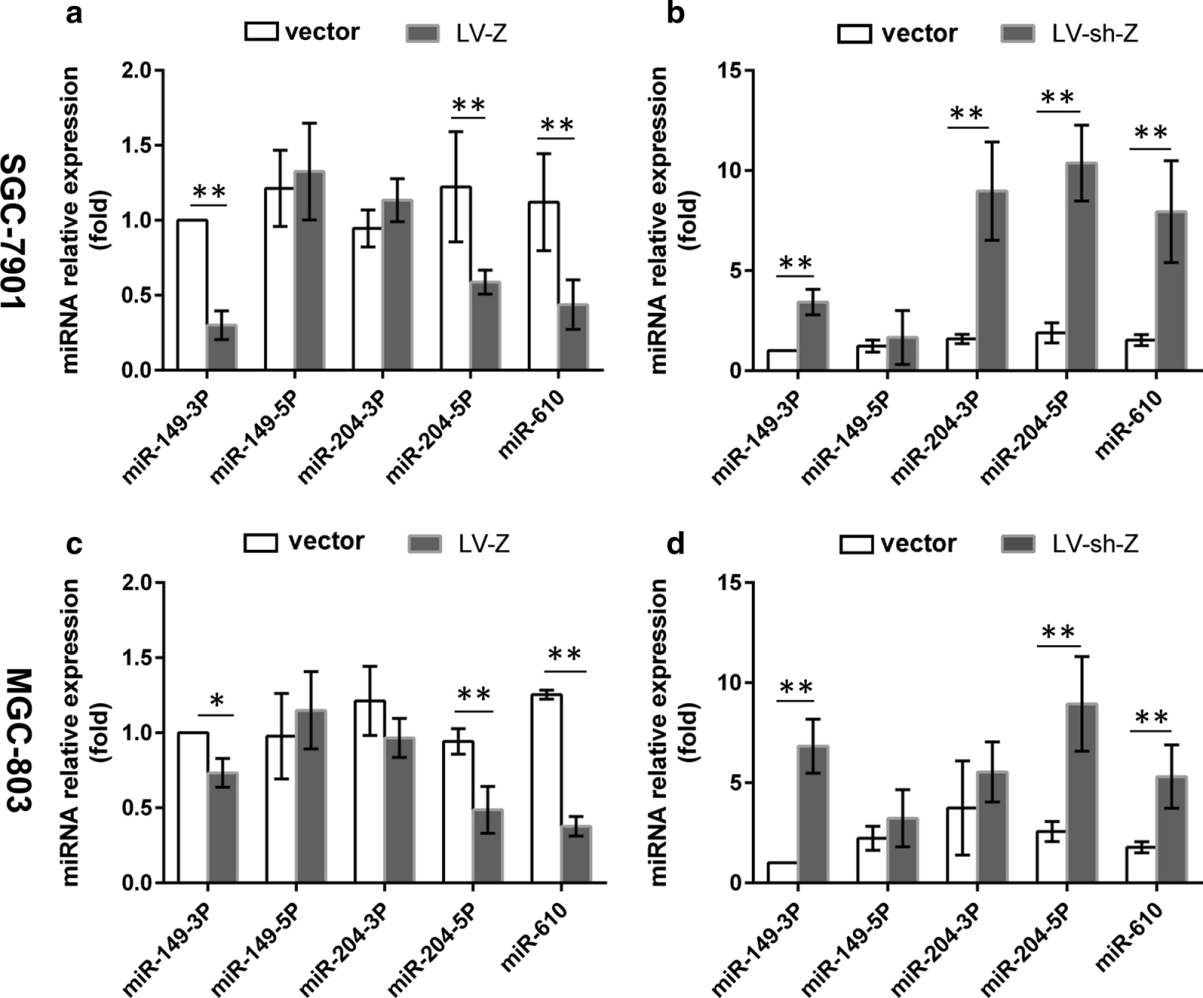
Repression of the miRNAs by ZEB1-AS1. **a** The expression of miR-149-3p, -204-5p, -610 was inhibited in SGC-7901 cells transfected with LV-Z. **b** The expression of miR-149-3p, -204-3p, -204-5p, -610 was promoted in SGC-7901 cells transfected with LV-sh-Z. **c** The expression of miR-149-3p, -204-5p, -610 was inhibited in MGC-803 cells transfected with LV-Z. **d** The expression of miR-149-3p, -204-5p, -610 was promoted in MGC-803 cells transfected with LV-sh-Z. LV-Z: ZEB1-AS1 overexpressing lentiviruses; LV-sh-Z: ZEB1-AS1-shRNA lentiviruses; vector; lentivirus blank vector. *P < 0.05; **P < 0.01

To verify the conclusions derived from bioinformatics analysis, primers for the three miRNAs, including miR-610, -149-3p, -149-5p, -204-3p, and -204-5p, were designed to examine their expression profiles by qPCR in SGC-7091 and MGC-803 cells transfected with either ZEB1-AS1 overexpression lentiviruses (LV-Z) or ZEB1-AS1 silencing lentiviruses (LV-sh-Z). Among the miRNAs, the expression of miR-149-3p, miR-204-5p, and miR-610, but not miR-149-5p and miR-204-3p, consistently displayed a reverse alteration following gain- or loss-of ZEB1-AS1 (Fig. 2). This is in keeping with the bioinformatics predictions. The results suggest that ZEB1-AS1 could negatively regulate miR-149-3p, -204-5p, and -610 through a ceRNA network. Previous studies have demonstrated that miR-610 and miR-204 can inhibit GC cell proliferation, invasion, migration and epithelial-mesenchymal transition (EMT) [36, 40]. miR-149-3p has been shown to be involved in GC cell apoptosis, cell cycle, and viability repression [41]. Given that miR-149-3p can participate in GC invasion and migration [42], we decided to investigate it further.

### Clinical relevance of ZEB1-AS1 and miR-149-3p

**Fig. 3:**
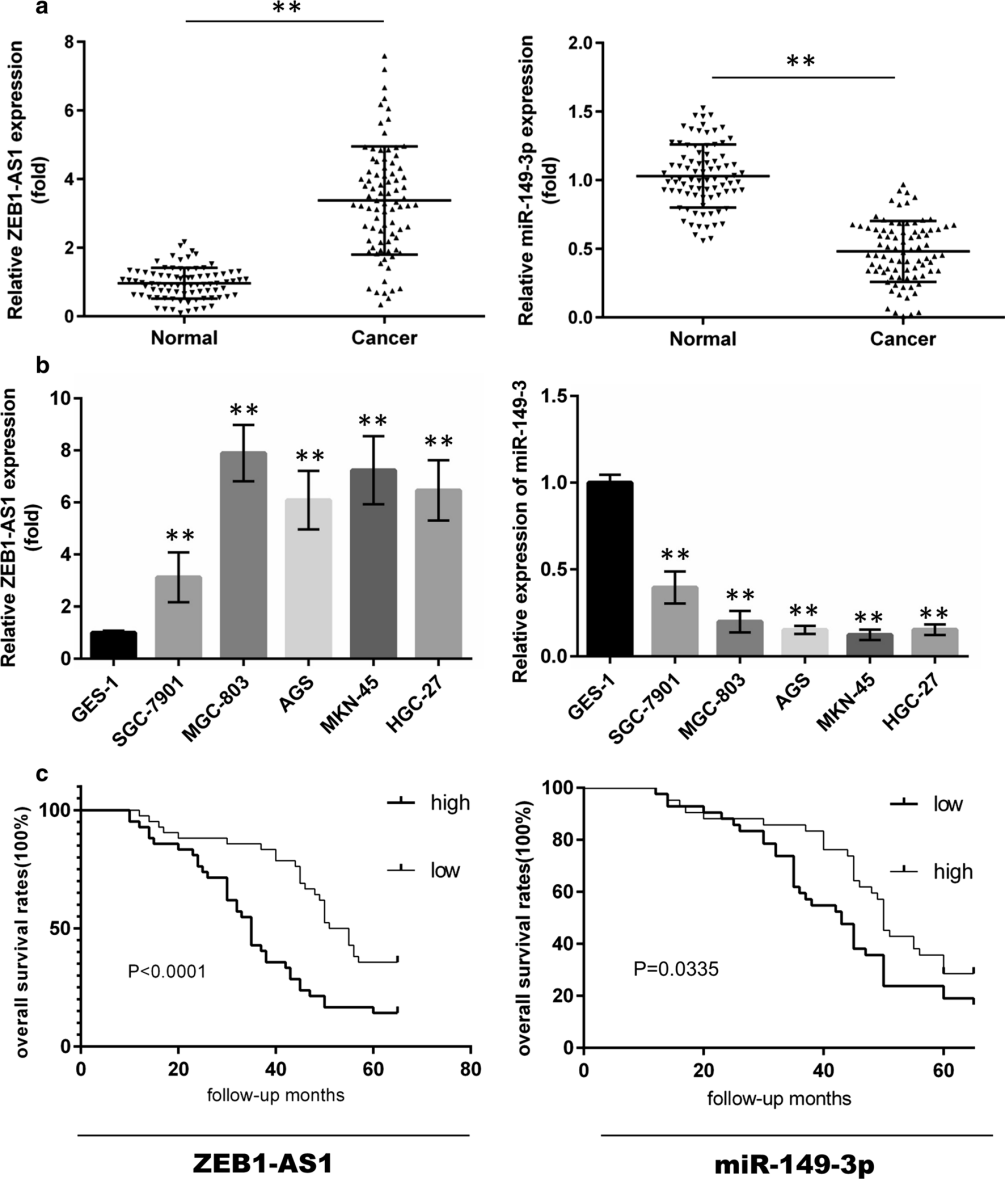
ZEB1-AS1 and miR-149-3p expression profiles in GC and their correlations with OS. **a** The expression of ZEB1-AS1 and miR-149-3p in 84 GC tissue samples (paired t-test). **b** The expression of ZEB1-AS1 and miR-149-3p in five GC and one GES-1 cell lines (one-way ANOVA). **c** High expression of ZEB1-AS1 or low expression of miR-149-3p is negatively associated with OS in GC (n = 84). OS: over survival. *P < 0.05; **P < 0.01

In order to examine expression levels of ZEB1-AS1 and miR-149-3p in GC tissue (Fig. 3a) and cell samples (Fig. 3b), qPCR was performed. Ectopic expression of ZEB1-AS1 and miR-149-3p was observed. To assess the clinical significance related to ZEB1-AS1 and miR-149-3p, patients were placed into high or low expression groups according to the median ZEB1-AS1 or miR-149-3p expression value in GC tissue. Following statistical evaluation using the X^2^ test, ZEB1-AS1 expression was positively correlated with tumor size, lymph node metastasis, and TNM stage. By contrast, miR-149-3p expression was negatively associated with invasion depth and TMN stage (Table 1). Accordingly, whether these two gene expression indicators could be exploited for prognosis in GC was investigated using univariate and multivariate Cox regression analyses. Univariate analysis supported the observation that invasion depth, TMN stage and ZEB1-AS1 or miR-149-3p expression levels were associated with OS (Table 2). Notably, the results suggested that ZEB1-AS1 was an unfavorable factor (HR: 2.786; 95% CI 1.216–6.153; P: 0.011), and miR-149-3p a favorable one (HR: 0.491; 95% CI 1.014–4.858; P: 0.041) in GC patients. Moreover, multivariate analysis indicated that ZEB1-AS1 and miR-149-3p expression, as well as TNM stage, were independent prognostic factors. Supportive of this conclusion, analysis using the Kaplan–Meier test showed that patients with high expression of ZEB1-AS1 or low expression of miR-149-3p were likely to receive a poor prognostic outcome (Fig. 3c).

**Table 2:** Uni- and multivariate analysis of prognostic predictors in gastric patients

Variable	Univariate analysis		Multivariate analysis	
	HR (95% CI)	P-value	HR (95% CI)	P-value
Age (≥ 60)	1.004 (0.985–1.023)	0.697		
Gender (male)	1.069 (0.649–1.762)	0.793		
Differentiation (poor)	1.207 (0.735–1.980)	0.458		
Lymph node metastasis (yes)	1.382 (1.017–2.783)	0.276		
Tumor size (> 5 cm)	1.303 (1.064–3.886)	0.375		
Invasion depth (T3–T4)	2.639 (1.561–4.459)	0.015*	2.235 (1.139–4.132)	0.111
TNM stage (III–IV)	5.232 (2.943–9.303)	0.001*	3.929 (2.115–7.299)	0.037*
miR-143-3p (high)	0.251 (0.039–2.176)	0.025*	0.491 (1.014–4.858)	0.041*
ZEB1-AS1 (high)	3.740 (1.636–4.589)	0.003*	2.786 (1.216–6.153)	0.011*

*HR* hazard rate, *CI* conscience interval

*P < 0.05

### Diagnostic value of ZEB1-AS1 and miR-149-3p

**Fig. 4:**
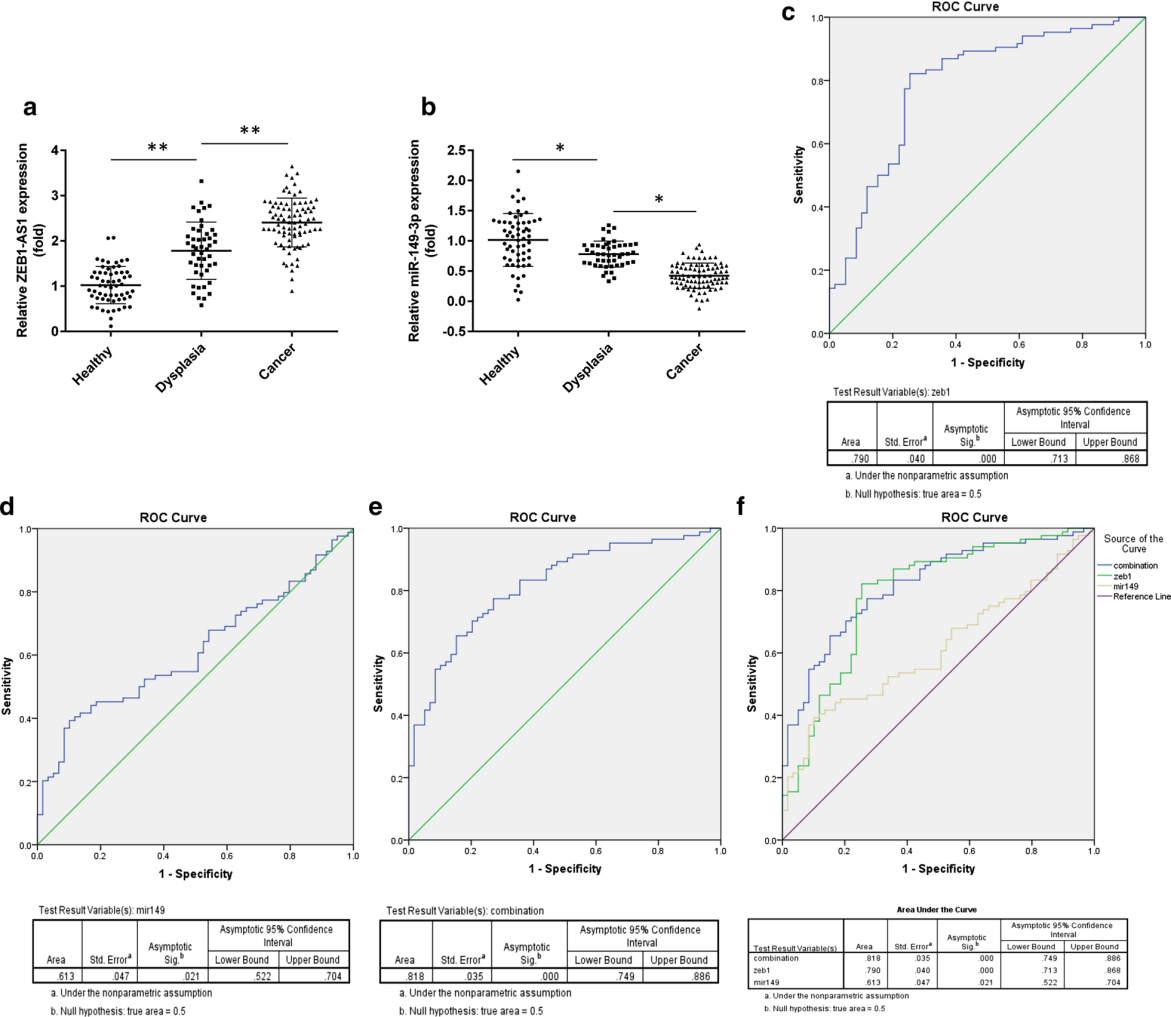
Early diagnostic value of ZEB1-AS1 and miR-149-3p. **a**, **b** The expression profiles of ZEB1-AS1 and miR-149-3p in 47 precancerous lesions, 59 healthy control samples and 84 GC specimens (one-way ANOVA). **c**–**e** The ROC curve of ZEB1-AS1, miR-149-3p and ZEB1-AS1 combined with miR-149-3p for distinguishing GC patients from healthy participants. **f** The three ROC curves are displayed together. *ROC* receiver operating characteristic. *P < 0.05; **P < 0.01

To ascertain the capacity of the two indicators to act as markers for early diagnosis of GC, validation of the expression levels of the two molecules was investigated by qPCR in 47 precancerous lesion, and 59 healthy control samples. Together with 84 GC patients, ZEB1-AS1 expression displayed a gradual increase from the healthy control to precancerous group and then to the cancer group (Fig. 4a). Nevertheless, a reverse trend was observed in miR-149-3p expression (Fig. 4b). Based on this validation, we propose that these two indicators might be valuable for the early diagnosis of GC. To verify this theory, we plotted a ROC curve using the PCR data from 59 healthy controls and 84 GC patients. According to the largest Youden’s index value, the following ROC curve outcomes are listed: the area under the curve (AUC) relating to ZEB1-AS1 reached 0.790. Sensitivity and specificity were 82.1% and 79.2%, respectively (Fig. 4c). The AUC of miR-149-3p was 0.613, along with 39.3% sensitivity and 89.8% specificity (Fig. 4d). The combined AUC value of ZEB1-AS1 and miR-149-3p was 0.818, with 77.4% sensitivity and 72.9% specificity (Fig. 4e). These data revealed that ZEB1-AS1 is more likely to efficiently distinguish GC cases than miR-149-3p, but that the combined effects of the two indicators provides optimal results.

### GC cell proliferation, migration and invasion were limited by miR-149-3p

**Fig. 5:**
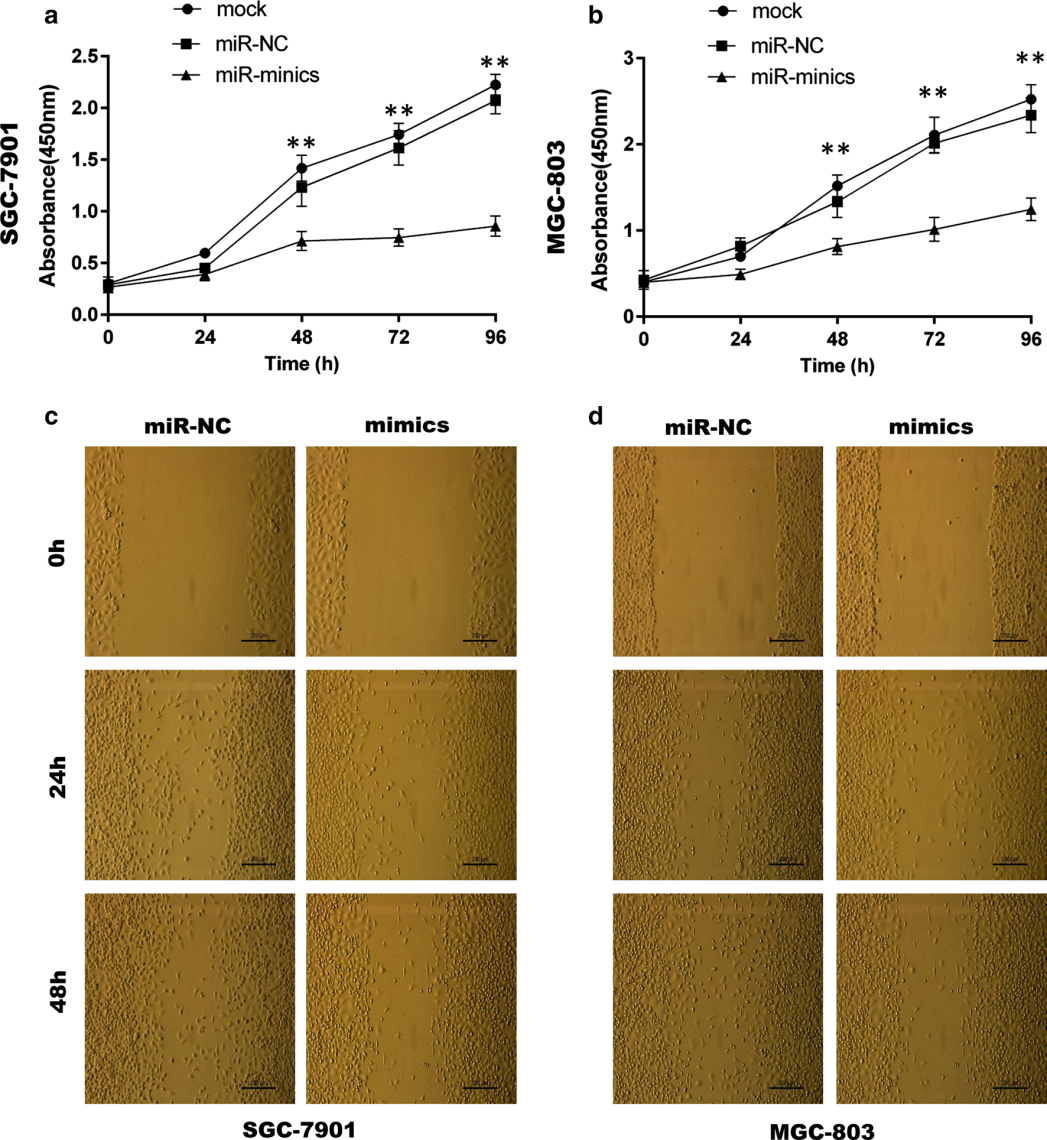
MiR-149-3p suppressed GC cell proliferation and migration. **a**, **b** The CCK-8 assays were performed in SGC-7901 and MGC-803 cells transfected with mimics or miR-NC (Two-way ANOVA). **c**, **d** The wound healing assays were performed in SGC-7901 and MGC-803 cells transfected with mimics or miR-NC. Mimics: 149-3p mimics; miR-NC: miR-149-3p negative control. *P < 0.05; **P < 0.01

**Fig. 6:**
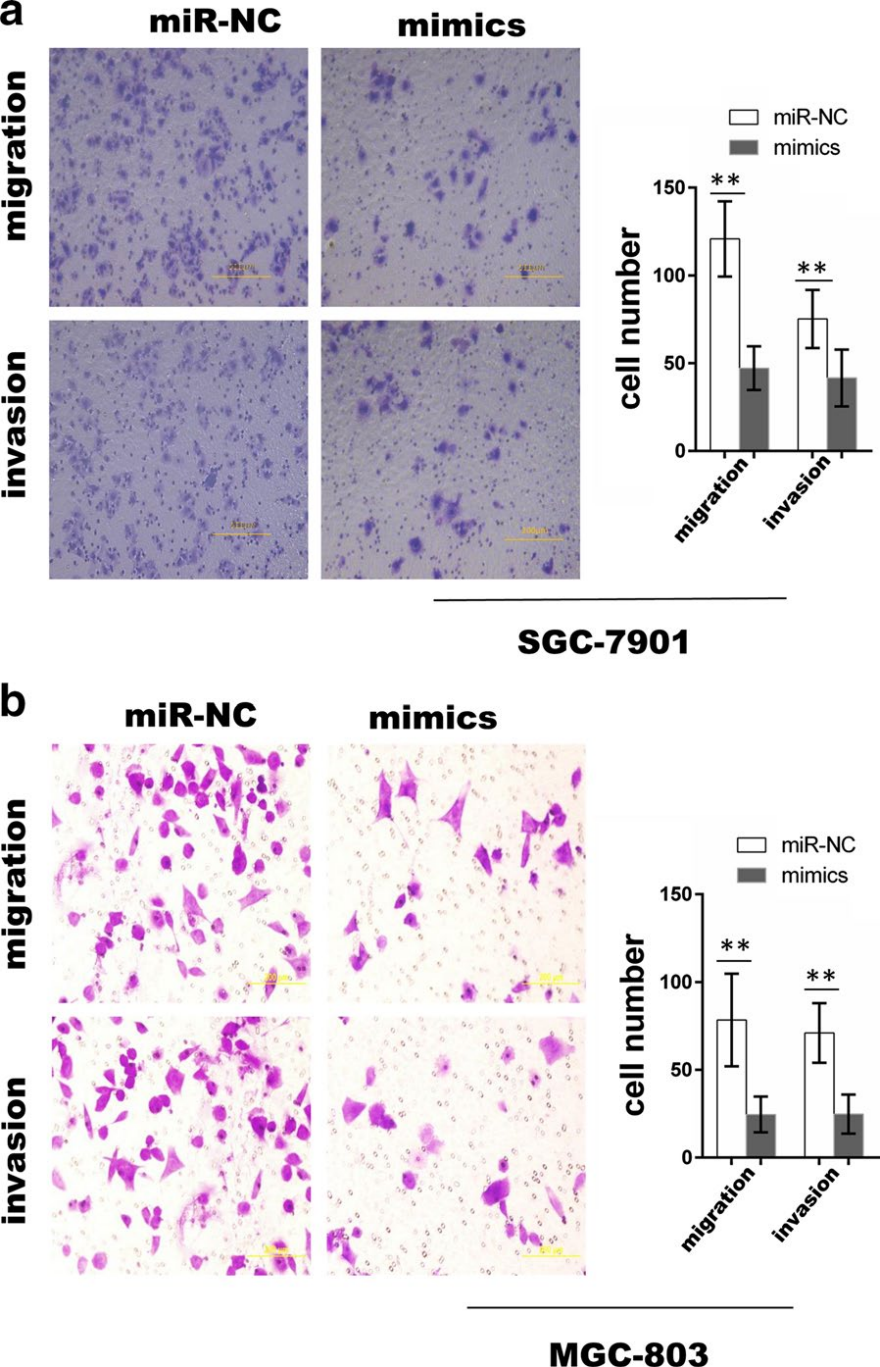
MiR-149-3p suppressed GC cell migration and invasion. **a**, **b** The transwell migration and invasion assays were performed in SGC-7901 and MGC-803 cells transfected with mimics or miR-NC (unpaired t-test). *P < 0.05; **P < 0.01

For functional assays, transfection of cells with a moderate dose of miR-149-3p mimics was found to lead to a specific decrease in miR-149-3p levels in SGC-7901 and MGC-803 cell lines (Additional file 1: Fig. S1). The miR-149-3p mimics were then transfected into the two GC cell lines with cell proliferation, wound healing and transwell assays being performed. The results showed that overexpression of miR-149-3p markedly limited GC cell viability (Fig. 5a, b) and suppressed their migratory and invasive properties (Figs. 5c, d, 6) in comparison to miR-NC.

### ZEB1-AS1 acts as a ceRNA network upon interaction with miR-149-3p

**Fig. 7:**
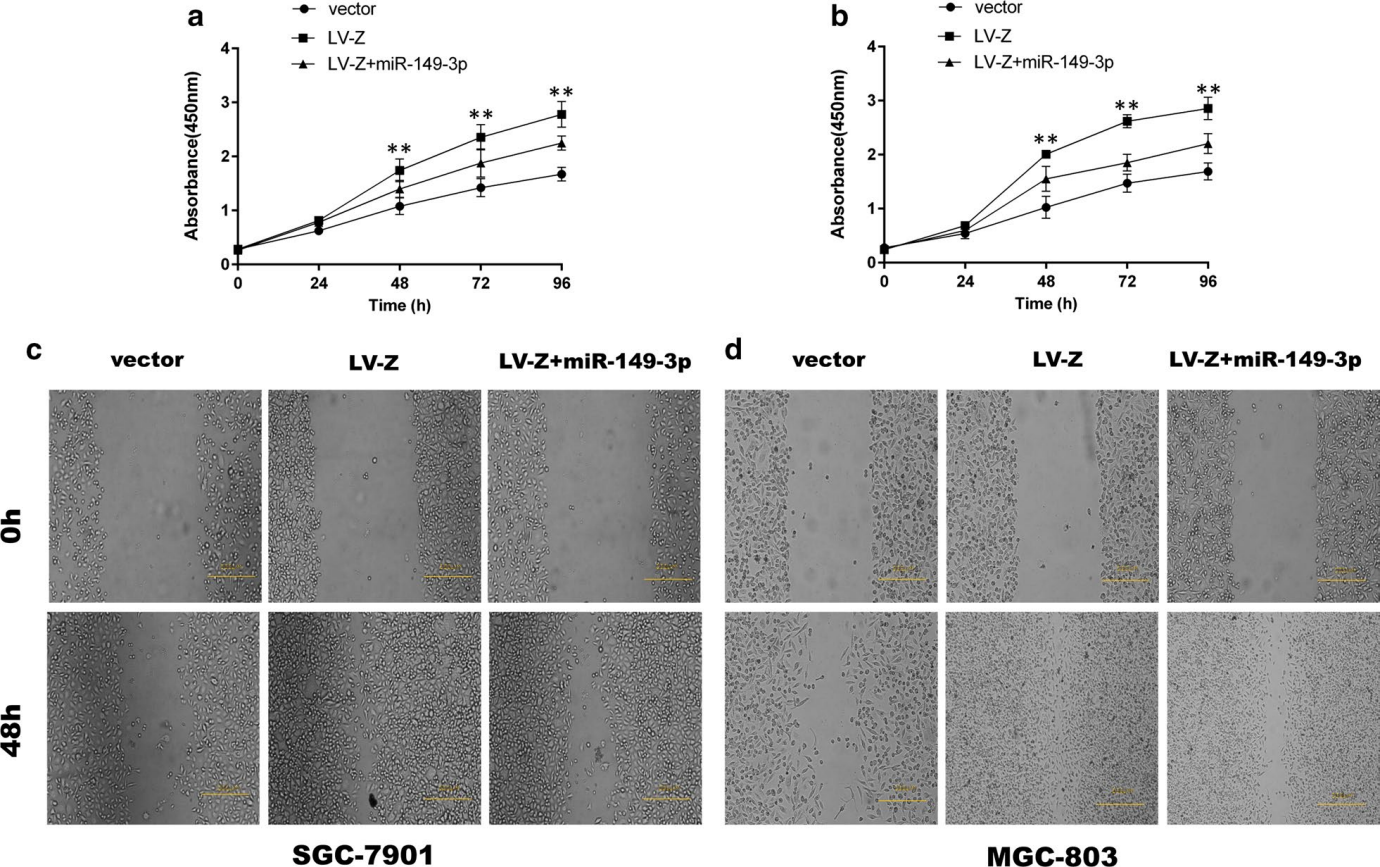
MiR-149-3p partly eliminated GC cell proliferation and migration induced by ZEB1-AS1. **a**, **b** ZEB1-AS1-induced cell proliferation was partly abrogated by miR-149-3p (Two-way ANOVA). **c**, **d** ZEB1-AS1-induced cell migration was partly abrogated by miR-149-3p. miR-149-3p: miR-149-3p mimics. *P < 0.05; **P < 0.01

**Fig. 8:**
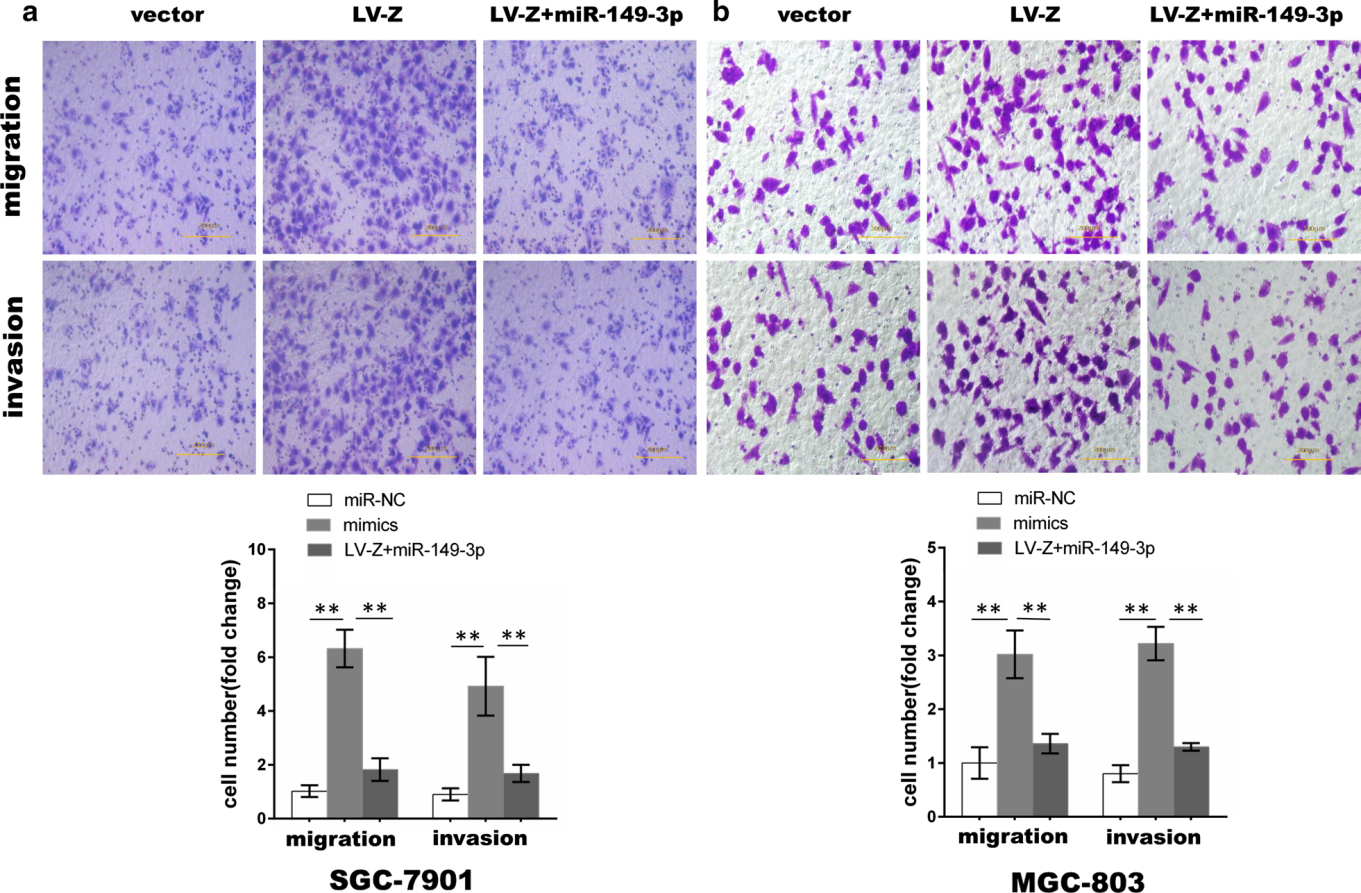
MiR-149-3p partially abolished GC cell migration and invasion induced by ZEB1-AS1. **a**, **b** ZEB1-AS1-induced cell migration and invasion were partly abrogated by miR-149-3p (Two-way ANOVA). *P < 0.05; **P < 0.01

To determine whether miR-149-3p can abrogate the effect of ZEB1-AS1, cells stably overexpressing ZEB1-AS1 were transfected with miR-149-3p mimics in order to perform cell proliferation, wound healing and transwell assays. As expected and confirmed through the cell proliferation assay, although ZEB1-AS1 overexpression evidently increased the proliferative capability of GC cells, miR-149-3p partly eliminated the effects induced by ZEB1-AS1 overexpression (Fig. 7a, b). Similar results were observed from the wound healing (Fig. 7c, d) and transwell assays (Fig. 8), further suggesting that miR-149-3p overexpression partially reverses ZEB1-AS1-induced cell migration and invasion.

**Fig. 9:**
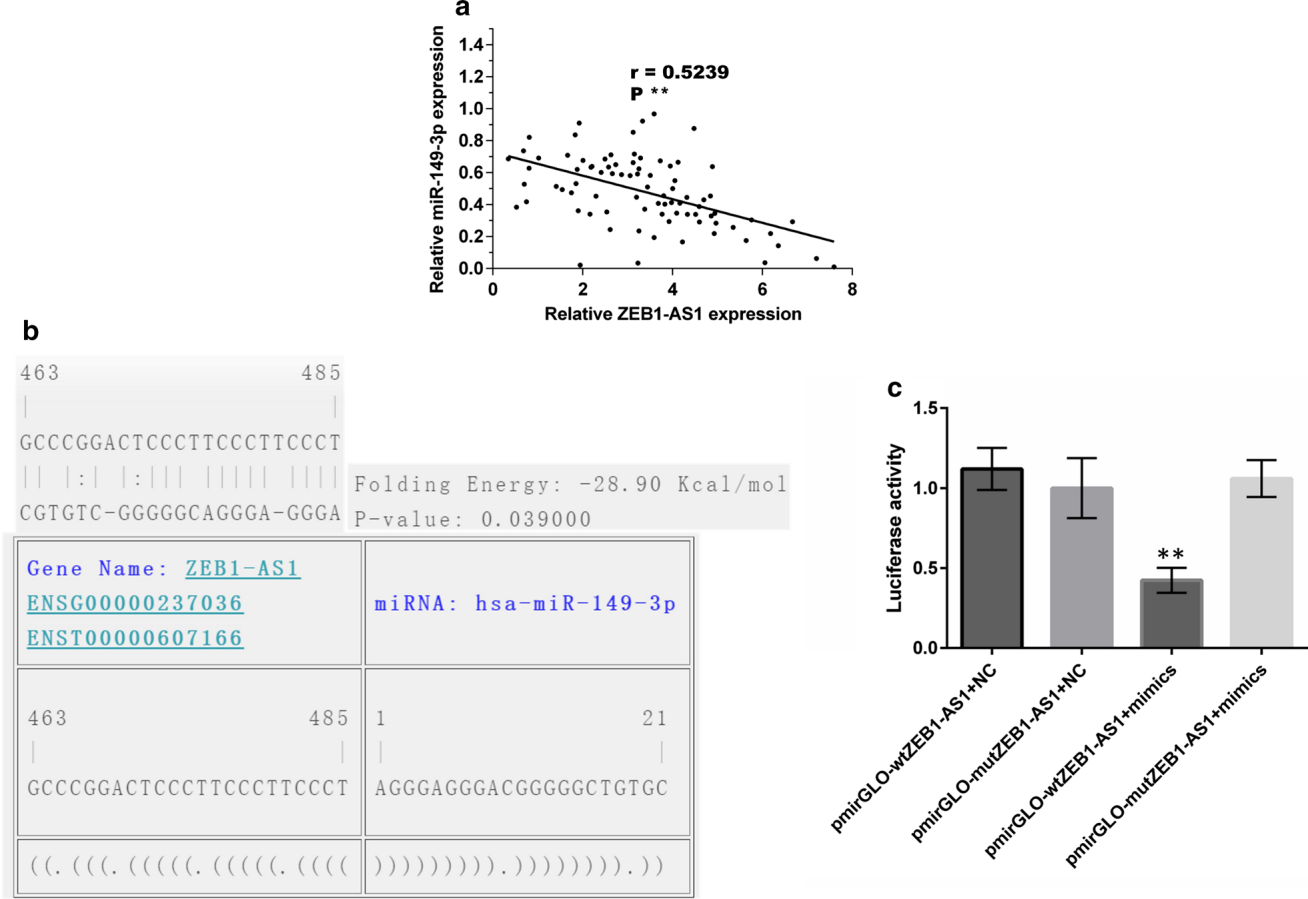
ZEB1-AS1 can bind to miR-149-3p. **a** A negative correlation between miR-149-3p and ZEB1-AS1 in 84 GC tissues was verified. **b** The putative miR-149-3p binding site in ZEB1-AS1 was predicted using RNA22 software. **c** Luciferase activity of ZEB1-AS1 in wild or mutant type (n = 3, one-way ANOVA). Mimics: miR-149-3p mimics; NC: miR-149-3p negative control. *P < 0.05; **P < 0.01

In vitro assays demonstrated that ZEB1-AS1 can sequester miR-149-3p to form a ceRNA network. The correlation between ZEB1-AS1 and miR-149-3p in 84 GC samples were further verified in order to more clearly demonstrate the formation of this network. Spearman’s correlation analysis indicated that there was a negative correlation between ZEB1-AS1 and miR-149-3p (R = 0.5239; P < 0.001) (Fig. 9a). Furthermore, a putative miR-149-3p binding site in ZEB1-AS1 was predicted using RNA22 version 2.0 tool (https://www.rna-seqblog.com/rna22-version-2-0-mirna-mre-predictions/) (Fig. 9b). Luciferase reporter vectors (pmirGLO) carrying wild type (wtZEB1-AS1) or mutant type ZEB1-AS1 (mutZEB1-AS1) molecules were constructed accordingly. Following co-transfection of the luciferase reporter vectors with miR-149-3p mimics or miR-NC, miR-149-3p mimics were observed to notably increase the luciferase activity of wtZEB1-AS1 but not that of mutZEB1-AS1. By comparison, miR-NC lacked the ability to exert such an effect (Fig. 9c). These results demonstrate that ZEB1-AS1 can competitively bind to miR-149-3p to form a ceRNA network.

## Discussion

It is well recognized that genes don’t work in isolation, but rather communicate with one other, thereby forming regulatory networks. LncRNAs, which are newly described and emerging regulatory elements, appeal to researchers as they participate in gene regulation in multiple manners. This includes engaging in competitive binding of specific miRNAs in order to form ceRNA networks. LncRNA-mediated sequestering of miRNAs could result in the increased expression of mRNAs which share miRNA Response Elements (MREs) with lncRNAs [43, 44]. Thus, the specific, common miRNAs-mediated crosstalk between lncRNAs and mRNAs weaves a competitive endogenous network [44], which extends the current dimensions and understanding of gene regulation. ZEB1-AS1 attracted our attention due to the fact that it can function as a tumor promoter in several cancers in miRNA-mediated manners [32–34]. Therefore, it is rational to hypothesize that ZEB1-AS1 could be involved in a miRNA-regulated ceRNA network in GC.

In the present study and by performing bioinformatics analysis, we speculate that ZEB1-AS1 can interplay with eight down-regulated miRNAs in GC. Among these miRNAs, the anti-tumor effects of miR-610, -204, and -149 on GC have been previously described, thereby supporting the reliability of our bioinformatics prediction. In addition, enrichment analysis for common target genes of the three miRNAs was performed. The enriched pathways included the PI3 K-Akt, MAPK, and Ras signaling pathways, which are explicitly related to GC progression and metastasis [45–47]. Hence, we theorized that ZEB1-AS1 can indirectly modulate these crucial pathways by sequestering the three miRNAs. In agreement with the bioinformatics conclusion, in vitro assays demonstrating the effect of gaining or losing ZEB1-AS1 indicated that it could regulate the expression of miR-149-3p, miR-204-5p and miR-610. Currently, our findings suggest that ZEB1-AS1 may sequester these three miRNAs to form a ceRNA network in GC. This finding is in keeping with previous research results. For example, the SNHG1 lncRNA sequestered miR-302/372/373/520 and consequently activated TGFBR2 and RAB11A in invasive pituitary tumors [48], while DANCR acted as a decoy for miR-335-5p and miR-1972, thus promoting ROCK1-associated proliferation and metastasis in osteosarcomas [49]. Our study extends the mechanism by which ZEB1-AS1 exerts its effect in GC. We furthermore infer that ZEB1-AS1 can act as a key therapeutic target in GC. In addition, in vitro assays showed that the expression patterns of miR-149-5p and miR-204-3p were variable and did not always match that of ZEB1-AS1. This implies that bioinformatics predictions need to be confirmed by additional tests.

The role of miR-149-3p in tumors remains controversial. For example, Bellazzo et al. report that it facilitates the aggressiveness of tumor cells in prostate cancer, bladder carcinoma, and breast carcinoma, to name a few [50]. However, Yang et al. observed that miR-149-3p blocks cell proliferation, migration, and invasion in bladder cancer [42]. Furthermore, its effects on GC cell proliferation, migration, and invasion are not fully elucidated. The exact function(s), underlying mechanism(s), and clinical significance relating to ZEB1-AS1 and miR-149-3p in GC therefore deserves further clarification.

In the present study, functional assays confirmed that miR-149-3p could suppress GC cell proliferation, migration, and invasion, thus indicating that miR-149-3p acts as a tumor repressor in GC. Its role in oncogene or tumor suppression was, however, found to be dependent on the type of tumor present. Quantitative real-time PCR analysis of ZEB1-AS1 and miR-149-3p first showed that the up-regulation of ZEB1-AS1 was accompanied by down-regulation of miR-149-3p in GC tissue. This observation was supported by the negative correlation found between the two factors through Pearson analysis. We speculated that the two factors could exert opposing functions in GC. Moreover, the in vitro malignant behaviors (i.e., proliferation, invasion, and migration) induced by ZEB1-AS1 could be abrogated by miR-149-3p. The luciferase reporter assay consistently indicated ZEB1-AS1 was directly bound by miR-149-3p. These results further confirm that, interacting with miR-149-3p, ZEB1-AS1 can form a ceRNA network. What is more, we theorized that ZEB1-AS1 can modulate GC occurrence, progress, and metastasis through a ZEB1-AS1/miR-149-3p axis; a thought reinforced by the outcomes from related studies. For example, LINC01088 targets miR-24-1-5p to inhibit ovarian epithelial cell tumor [51], while the lncRNA HNF1A-AS1 represses the miR-34a/SIRT1/p53 axis to promote colon cancer metastasis [52].

In addition, our study has revealed that high expression of ZEB1-AS1 or low expression of miR-149-3p could serve as independent indicators of an unfavorable prognosis in GC patients. Similarly, an increasing number of studies support the belief that ZEB1-AS1 might be attributed to a poor prognosis in GC [19], colorectal cancer [53], gliomas [17], esophageal squamous cell carcinoma [18], and hepatocellular carcinoma [16]. To the best of our knowledge, no report has previously described miR-149-3p as a possible prognostic factor in GC; the implication thereof is indicated here for the first time. Furthermore, with reliable ROC curve analyses and AUC outputs with good sensitivity and specificity, we conclude that ZEB1-AS1 could potentially be used as a marker for the early diagnosis of GC. Diagnostic capacity might be improved should ZEB1-AS1 and miR-149-3p be used in combination. To our knowledge, our study is also the first to evaluate the early diagnostic ability of ZEB1-AS1 and miR-149-3p in GC.

## Conclusion

ZEB1-AS1 can interact with specific miRNAs to form a miRNA-mediated ceRNA network and, partly via a ZEB1-AS1/miR-149-3p axis, promote GC progression. ZEB1-AS1 and miR-149-3p might serve as promising biomarkers for GC prognosis and diagnosis, while the ZEB1-AS1/miR-149-3p axis could provide new insights into the treatment of GC.

## Additional files

**Additional file 1: Fig. S1.** The transfection efficiency of ZEB1-AS1 and miR-149-3p. A: The transfection efficiency of miR-149-3p mimics was confirmed by qPCR. B, C: The transfection efficiency of LV-Z and LV-sh-Z were confirmed by qPCR too. *P < 0.05; **P < 0.01.

**Additional file 2: Table S1.** The sequence information involved in the study.

**Additional file 3.** LV-ZEB1-AS1 sequence.

**Additional file 4.** The down-regulated miRNAs were obtained by screening the TCGA database.

**Additional file 5.** The miRNAs binding to ZEB1-AS1 were predicted using RNA22 software.

**Additional file 6.** 8 down-regulated miRNAs were selected after intersecting the two miRNA groups.

**Additional file 7.** 576 consensus genes targeted by miR-610, miR-149 and miR-204.

**Additional file 8.** The gene enrichment analysis of the 576 target genes.

## Abbreviations

GC: gastric cancer
ZEB1-AS1: LncRNA ZEB1 antisense 1
qPCR: quantitative real time polymerase chain reaction
miR-NC: miR-149-3p negative control
LV-Z: ZEB1-AS1 overexpressing lentiviruses
LV-sh-Z: ZEB1-AS1-shRNA lentiviruses
wtZEB1-AS1: ZEB1-AS1 wild type
mutZEB1-AS1: ZEB1-AS1 mutant type
CCK-8: Cell Counting Kit-8
ceRNA: competing endogenous RNAS
ROC curve: receiver operating characteristic curve analysis
OS: overall survival
AUC: the area under the curve
HR: hazard rate
CI: conscience interval
MREs: miRNA Response Elements
