# Correction to: ZEB1‑AS1 initiates a miRNA‑mediated ceRNA network to facilitate gastric cancer progression

**DOI:** 10.1186/s12935-019-0797-y

**Published:** 2019-04-11

**Authors:** Ming-Hui Ma, Jia-Xiang An, Cheng Zhang, Jie Liu, Yu Liang, Chun-Dong Zhang, Zhen Zhang, Dong-Qiu Dai

**Affiliations:** 1 Department of Gastroenterological Surgery, The Fourth Affiliated Hospital of China Medical University, Shenyang, 110032 China; 2 Science Experiment Center, China Medical University, Shenyang, 110122 China

## Correction to: Cancer Cell Int (2019) 19:27 10.1186/s12935-019-0742-0

After publication of the original article [1], the authors have notified us of 5 errors which are highlighted below. The authors apologize for any inconvenience.

1. “ca-199 (tissue) (U/mL)” in Table 1 was incorrectly presented and should be replaced with “ca-199 (serum) (U/mL)”.
2. “miR-143-3p” in Table 2 was a mistake in spelling and should be replaced with “miR-149-3p”.
3. We have corrected the 48h representation in Fig. 5.

**Fig. 5:**
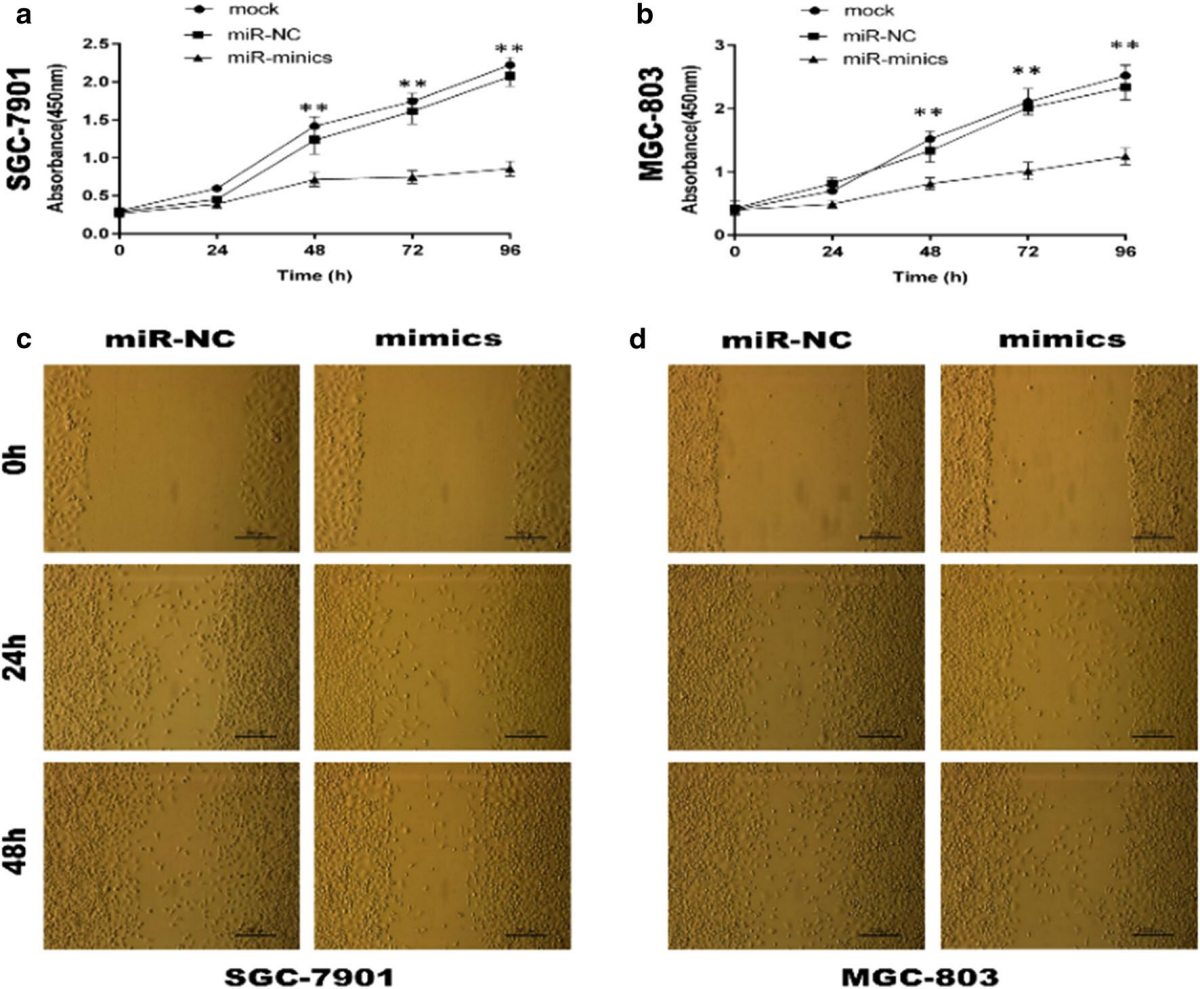
MiR-149-3p suppressed GC cell proliferation and migration.** a**,** b** The CCK-8 assays were performed in SGC-7901 and MGC-803 cells transfected with mimics or miR-NC (Two-way ANOVA).** c**,** d** The wound healing assays were performed in SGC-7901 and MGC-803 cells transfected with mimics or miR-NC. Mimics: 149-3p mimics; miR-NC: miR-149-3p negative control. *P < 0.05; **P < 0.01
4. We have corrected the cell migration vector representation and the cell invasion LV-Z+miR-149-3p representation in Fig. 8.

**Fig. 8:**
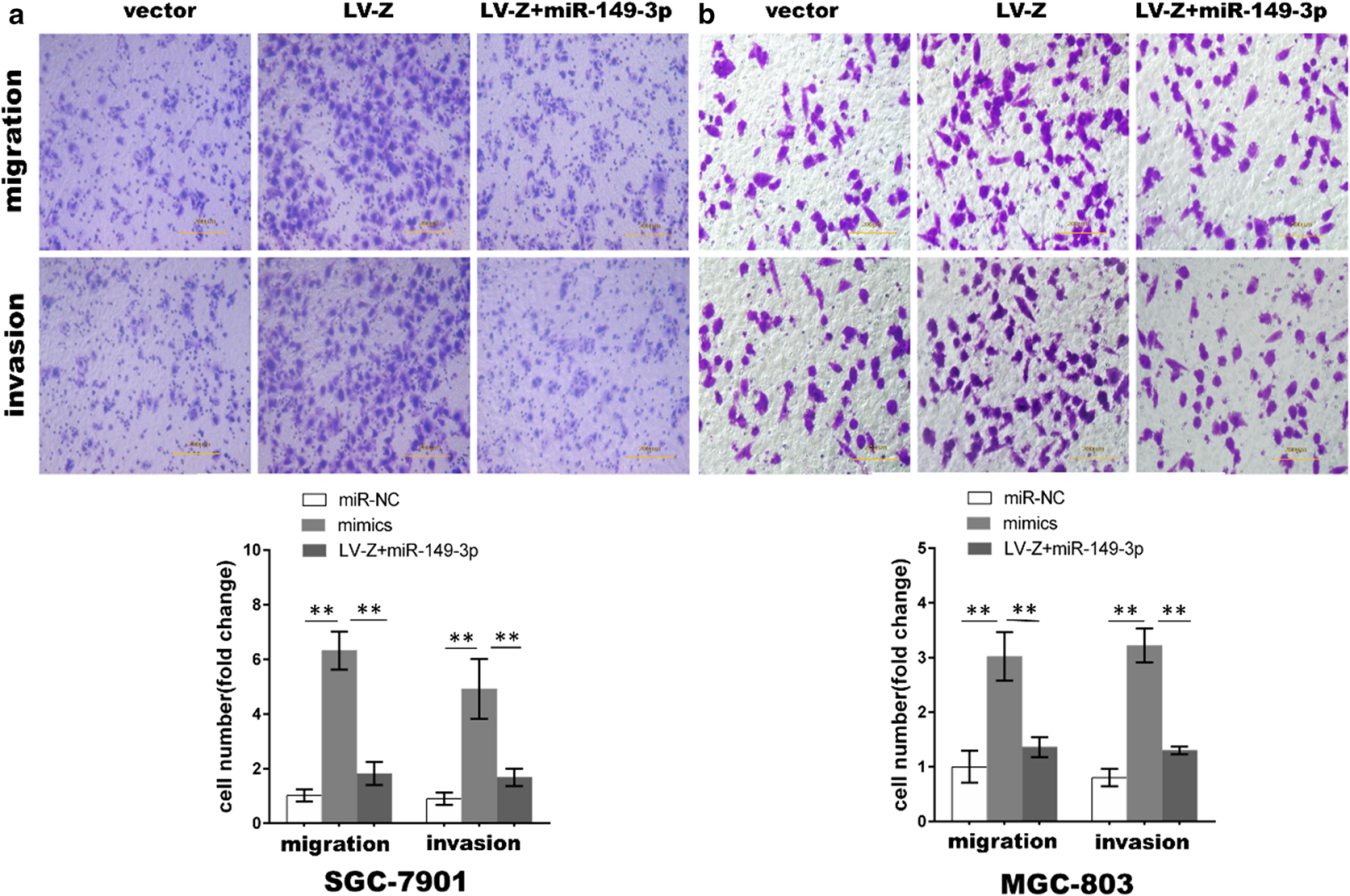
MiR-149-3p partially abolished GC cell migration and invasion induced by ZEB1-AS1.** a**,** b** ZEB1-AS1-induced cell migration and invasion were partly abrogated by miR-149-3p (Two-way ANOVA). *P < 0.05; **P < 0.01
5. The primer sequences with red color were out-of-order for the wrong type setting of Microsoft Word. Accordingly, we corrected it.

## Additional file

**Additional file 2: Table S1.** The sequence information involved in the study (Wrong). The sequence information involved in the study (Revised).
