# Supplementary figures and images for: ZEB1-AS1 initiates a miRNA-mediated ceRNA network to facilitate gastric cancer progression
Source: Cancer Cell Int. 2019 Feb 6;19:27. doi: 10.1186/s12935-019-0742-0 (PMC6364449; doi:10.1186/s12935-019-0742-0)

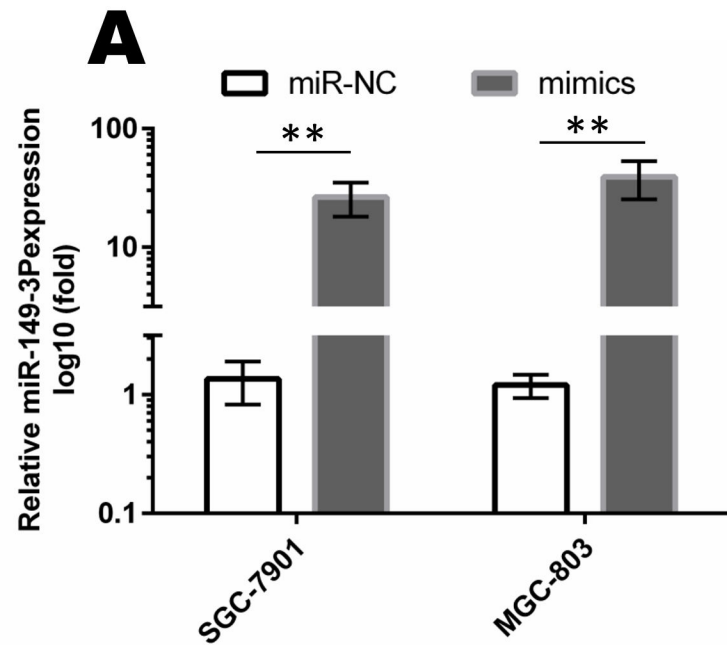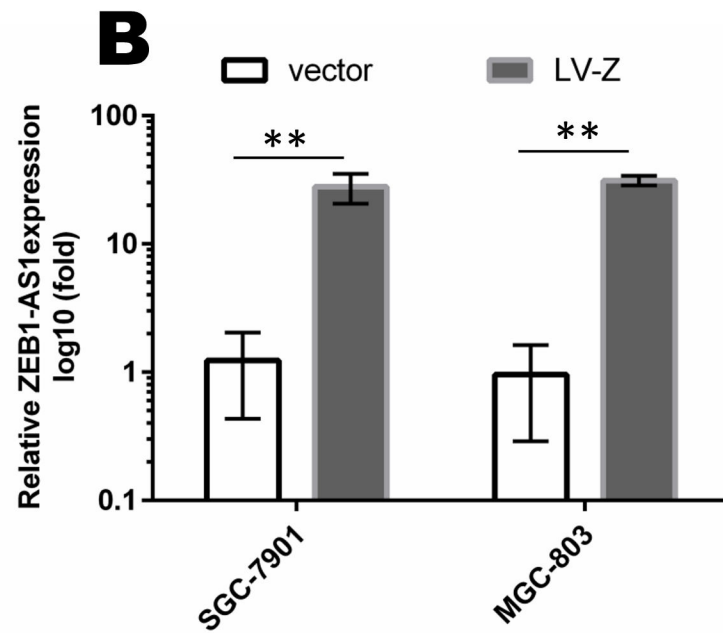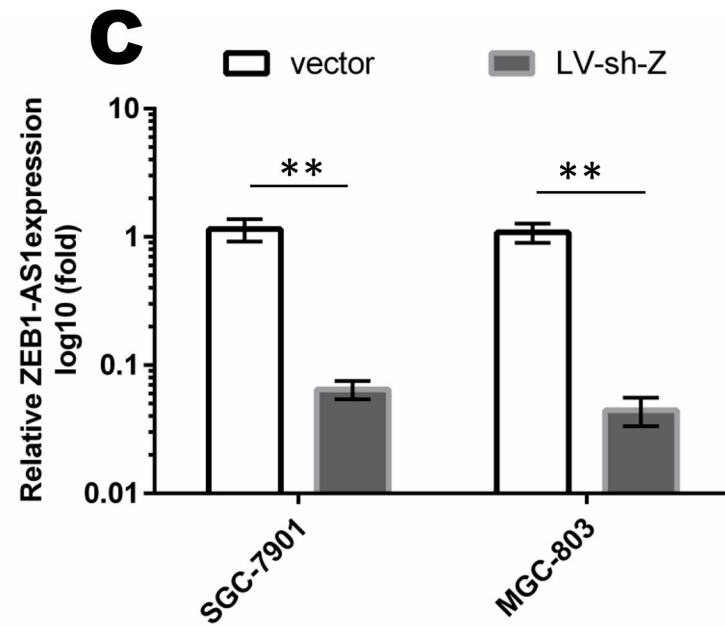

Supplement: Supplementary file 1 — Additional file 1: Fig. S1. The transfection efficiency of ZEB1-AS1 and miR-149-3p. A: The transfection efficiency of miR-149-3p mimics was confirmed by qPCR. B, C: The transfection efficiency of LV-Z and LV-sh-Z were confirmed by qPCR too. *P < 0.05; **P < 0.01. [file 12935_2019_742_MOESM1_ESM.pdf]
