# Supplementary material for: ZEB1-AS1 initiates a miRNA-mediated ceRNA network to facilitate gastric cancer progression
Source: Cancer Cell Int. 2019 Feb 6;19:27. doi: 10.1186/s12935-019-0742-0 (PMC6364449; doi:10.1186/s12935-019-0742-0)
Supplement: Supplementary file 2 — Additional file 2: Table S1. The sequence information involved in the study. [file 12935_2019_742_MOESM2_ESM.pdf]

Table S1. The sequence information involved in the study

| name | sequence |
|---|---|
| LV-sh-Z | Sense:tCGAATCAGGTCATAGACTAttcaagagaTAGTCTATGACCTGATTCGttttttc |
| | Antisense:tcgagaaaaaaCGAATCAGGTCATAGACTAtctcttgaaTAGTCTATGACCTGATTCGa |
| miR-149-3P mimics | Sense:5’-AGGGAGGGACGGGGGCUGUGC-3, |
| | Antisense: 5’-ACAGCCCCCGUCCCUCCCUUU-3’ |
| miR-149-3p inhibiter | 5’-GCACAGCCCCCGUCCCUCCCU-3’ |
| miR-149-3p-NC | Sense:5’-UGAGCUAAAUGUGUGCUGGGA-3’; |
| | Antisense 5’-CCAGCACACAUUUAGCUCAUU-3’ |
| ZEB1-AS1 primer | Sense:5’- GAACCGGGATGGGAAGTGAC -3’; |
| | Antisense:5’-GCAAGCGGAACTTCTAGCCT -3’ |
| miR-149-3p primer | Sense: 5'- GAACCGGGATGGGAAGTGAC -3' |
| | Antisense: 5'- GCAAGCGGAACTTCTAGCCT-3' |
| miR-149-5p primer | Forward: 5’-AGGGAGGGACGGGGGCT-3’ |
| mir-204-3p primer | Forward: 5’-TCTGGCTCCGTGTCTTCACTC-3’ |
| miR-204-5p primer | Forward: 5’-GCTGGGAAGGCAAAGGGAC-3’ |
| miR-610 primer | Forward: 5’-CGTTCCCTTTGTCATCCTATGCCT-3’ |
| | Forward: CGTGAGCTAAATGTGTGCTGGGA |
| GAPDH primer | Sense: 5’- CCTGGCACCCAGCACAAT -3’ |
| | Antisense: 5’- GGGCCGGACTCGTCATAC -3’ |
| U6 primer | Sense: 5’-CTCGCTTCGGCAGCACA-3’ |
| | Antisense: 5’-AACGCTTCACGA ATTTGCGT-3’ |
