# Supplementary material for: ZEB1-AS1 initiates a miRNA-mediated ceRNA network to facilitate gastric cancer progression
Source: Cancer Cell Int. 2019 Feb 6;19:27. doi: 10.1186/s12935-019-0742-0 (PMC6364449; doi:10.1186/s12935-019-0742-0)
Supplement: Supplementary file 3 — Additional file 3. LV-ZEB1-AS1 sequence. [file 12935_2019_742_MOESM3_ESM.pdf]

LV-ZEB1-AS1 sequence

CTTACACCTAGAGGCTCTCGCTCTACGGCCGGAACCTTGTTGCTAGGGACCGGGCGGTTTGCGG
CAACCGTGGGCACTGCTGAATTTGAATTGAGGGGCGAGGGAAAAGTTTTCCTCAGGTGTGGTG
GGGAGAGGGAGGCGGATGCCGGGAAACCGTAGGGACGCGGTCAGAAAGGCGACGGGCTGTC
GGAGTTGGAAAGGGACGCCTGGTTTCCCCCCAAGCGAACCGGGATGGGAAGTGACTTCAATGA
GATTGAACTTCAGCTGGATTGAAAGAGAGGCTAGAAGTTCCGCTTGCCAGCAGCCTCCTTAGTA
GAGCGGAATGAGTAATACCCACACGGTGCTTGTCTCACTTCCCCATCCGCACCCGGCCCTCACC
TGCTGTCACCTCGGCCTCCCACACCCGGTCCGCGCTCCCCGCCCTCTTCCTCGCGTAGAACCGT
GGGATCCTAGGTGGCAGGACTCAGAGCTAAGGTATCCACAGGCCATGAATTCCTTCCTAAATGA
GCGGTCATCGCCGTGCAGGACCTTAAGGCAAGAAGCATCGGCTGACAGATGTGATCTCTGAAC
CTGATAGATTGCTGATTTTATCTTATTTTATCCTTGACTTGGTACAAGTTTTGGGATTTCTGAAAAG
ACCATACAGATAACCACAAATATCAAGAAAGTCGTCTTCAGTATTAAGTAGAATTTAGATTTAGG
TTTCCTTCCTGCTTCCCACCTCCTTCGAATAAGGAAACGTCTTTGGGACCAACTTTATGGAATAA
ATAAGCTGAGCTGTATTTCAAGTAATATAGTTATAAATTAACAATGTAGCAGTTATTGATAGAGA
AATTGAGAAAACTGAAACGTGACCGGAGTATTGGAAATAACGTAGTACATCACCTAGCACAAT
GACACATAGTAGGTGCTCAATAAATTTATGCTTATAATTTTTGTCACTTCTATGGCAGGATTTTTTT
ATTAGGTTAAAATTATCTTTTAAACACCTTCCGGAATTTTAGAATATTCATTAATAATGTCTTCAAA
CCTTTCAACTGAAATAAATTTACAGCTGAAGTCTGATGATTTAAAGTTAGAAAGTTTAATCTTGAA
TATAAATGAACATTTTCTCTCCCACATTTTCTTGGGCATTTTGAGAAGTAAATGCGTTATTTATTG
GTCCATGAAATGTGACTGTAAATATTCTTTGCTATACATTATGTCTATATATCTGCATTCATCCTCA
ATGCCAAAACTAGAATCATTAGTCTTAATGATCATTTTAAGTACAGGCAGTCCTCGCTTTCCTTG
ATACCATGTTAACCGAAACTTGTGTATGTCAACACGGTGTCCTTGCTTTGCTTGGTTAAGTGTGA
GTTCTTCCTCCCTTTTTTTAAGAGTTGTACAATGTTTTTCAGTCGCCTACCGAATCAGGTCATAGA
CTATGGAATTGACCCCACCCCACCAACATTTTTACAGCTACCCTGATTTCTGACCAGAAAGGAA
AAAAAAACTTTCCAGCTCTATCACACATTTTACCTACTCTTAAACTTAGGAGGTATTACAAATAG
CATTTTCTCATGTTCTCTTTCTGGCCTGTACCTCCCTGCTAAGCTTCCTTCAGTGTTCATCCTCACC
TCATAGAGAGATGAAGTGAAGAGACAAACAGAAGTCATTTTCTTCCTTACTTTAGTGGTTTCTGG
TTTAGTTAGTTTGGGCCAAACTGTGGACAAGTACCTTTTCAGGTAACTTTTTTTTCTTATTTCTATG
TCCTCAACACCTAGTGGAGTACGTAGCCAATAGTAGATGCTTAATAAACATTTCTTAAATTAATA
TTGTTGACCTTTTCTGACCCTGTTCTTGACAGTAAGGTACATAATCTGCCTTCATCCCTTTAGTCCT
TAGGAACAGATAAAGTCATGGATATGAAAGTGATCACTGTCATTAATATCCACATTAAAATTGCT
CTTGATTTTAGTTTCTCCATAATCATTTTCCCTAAACAATGAACTCTGTTCACCTTTTTTTTTAAAAT
ATGCACAGTGAATATTACTGGTAGCCCAAATCTTCTAACATAAAATTTCCATTTTGTAAAAGCTTC
TGATAAGCATATATGTTATGAATTGAATGTTTGATTATTATACTTTAATATTCTTGAAAATATTGAT
ACCTGGACTGGAAAGAAAACAGACAAAAGTAAATCTCAGAATAAATTACTGCTTTAAACATGAA
