# Supplementary material for: Correction to: ZEB1‑AS1 initiates a miRNA‑mediated ceRNA network to facilitate gastric cancer progression
Source: Cancer Cell Int. 2019 Apr 11;19:95. doi: 10.1186/s12935-019-0797-y (PMC6458764; doi:10.1186/s12935-019-0797-y)
Supplement: Supplementary file 1 — Additional file 2: Table S1. The sequence information involved in the study (Wrong). The sequence information involved in the study (Revised). [file 12935_2019_797_MOESM2_ESM.docx]

Additional file 2: Table S1. The sequence information involved in the study **(Wrong)**

| name | sequence |
| --- | --- |
| LV-sh-Z | Sense: tCGAATCAGGTCATAGACTAttcaagagaTAGTCTATGACCTGATTCGttttttc |
|  | Antisense: tcgagaaaaaaCGAATCAGGTCATAGACTAtctcttgaaTAGTCTATGACCTGATTCGa |
| miR-149-3P mimics | Sense:5’-AGGGAGGGACGGGGGCUGUGC-3,  Antisense: 5’-ACAGCCCCCGUCCCUCCCUUU-3’ |
| miR-149-3p inhibiter | 5’-GCACAGCCCCCGUCCCUCCCU-3’ |
| miR-149-3p-NC | Sense:5’-UGAGCUAAAUGUGUGCUGGGA-3’;  Antisense 5’-CCAGCACACAUUUAGCUCAUU-3’ |
| ZEB1-AS1 primer | Sense:5’- GAACCGGGATGGGAAGTGAC -3’;  Antisense:5’-GCAAGCGGAACTTCTAGCCT -3’ |
| miR-149-3p primer | Sense: 5'- GAACCGGGATGGGAAGTGAC -3'  Antisense: 5'- GCAAGCGGAACTTCTAGCCT-3' |
| miR-149-5p primer | Forward: 5’-AGGGAGGGACGGGGGCT-3’ |
| mir-204-3p primer | Forward: 5’-TCTGGCTCCGTGTCTTCACTC-3’ |
| miR-204-5p primer | Forward: 5’-GCTGGGAAGGCAAAGGGAC-3’ |
| miR-610 primer | Forward: 5’-CGTTCCCTTTGTCATCCTATGCCT-3’ |
|  | Forward: CGTGAGCTAAATGTGTGCTGGGA |
| GAPDH primer | Sense: 5’- CCTGGCACCCAGCACAAT -3’  Antisense: 5’- GGGCCGGACTCGTCATAC -3’ |
| U6 primer | Sense: 5’-CTCGCTTCGGCAGCACA-3’  Antisense: 5’-AACGCTTCACGA ATTTGCGT-3’ |

Additional file 2: Table S1. The sequence information involved in the study **(Revised)**

| name | sequence |
| --- | --- |
| LV-sh-Z | Sense: tCGAATCAGGTCATAGACTAttcaagagaTAGTCTATGACCTGATTCGttttttc |
|  | Antisense: tcgagaaaaaaCGAATCAGGTCATAGACTAtctcttgaaTAGTCTATGACCTGATTCGa |
| miR-149-3P mimics | Sense:5’-AGGGAGGGACGGGGGCUGUGC-3 |
|  | Antisense: 5’-ACAGCCCCCGUCCCUCCCUUU-3’ |
| miR-149-3p inhibiter | 5’-GCACAGCCCCCGUCCCUCCCU-3’ |
| miR-149-3p-NC | Sense:5’-UGAGCUAAAUGUGUGCUGGGA-3’ |
|  | Antisense: 5’-CCAGCACACAUUUAGCUCAUU-3’ |
| ZEB1-AS1 primer | Sense:5’- GAACCGGGATGGGAAGTGAC -3’ |
|  | Antisense:5’-GCAAGCGGAACTTCTAGCCT -3’ |
| miR-149-3p primer | Sense: 5'- ACACTCCAGCTGGGAGGGAGGGACGGGGGCTG -3' |
|  | Antisense: 5'- CTCAACTGGTGTCGTGGA -3' |
| miR-149-3p primer | Forward: 5’-- AGGGAGGGACGGGGGCT -3' |
| miR-149-5p primer | Forward: 5'- TCTGGCTCCGTGTCTTCACTC -3 |
| mir-204-3p primer | Forward: 5’-GCTGGGAAGGCAAAGGGAC-3’ |
| miR-204-5p primer | Forward: 5’-CGTTCCCTTTGTCATCCTATGCCT-3’ |
| miR-610 primer | Forward: 5’-CGTGAGCTAAATGTGTGCTGGGA-3’ |
| GAPDH primer | Sense: 5’- CCTGGCACCCAGCACAAT -3’ |
|  | Antisense: 5’- GGGCCGGACTCGTCATAC -3’ |
| U6 primer | Sense: 5’-CTCGCTTCGGCAGCACA-3’ |
|  | Antisense: 5’-AACGCTTCACGA ATTTGCGT-3’ |
